# Electron and Hole Mobilities in Bulk Hematite from Spin-Constrained Density Functional Theory

**DOI:** 10.1021/jacs.1c13507

**Published:** 2022-03-03

**Authors:** Christian S. Ahart, Kevin M. Rosso, Jochen Blumberger

**Affiliations:** † Department of Physics and Astronomy, University College London, London WC1E 6BT, U.K.; ‡ Pacific Northwest National Laboratory, Richland, Washington 99354, United States

## Abstract

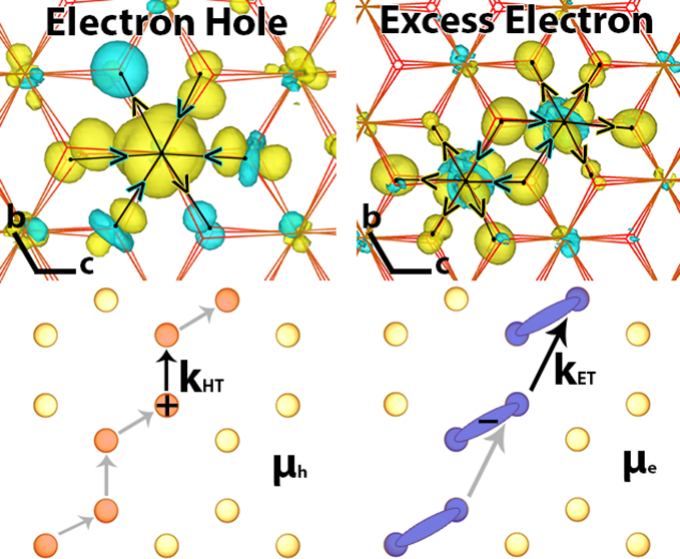

Transition metal oxide materials have attracted much attention for photoelectrochemical water splitting, but problems remain, e.g. the sluggish transport of excess charge carriers in these materials, which is not well understood. Here we use periodic, spin-constrained and gap-optimized hybrid density functional theory to uncover the nature and transport mechanism of holes and excess electrons in a widely used water splitting material, bulk-hematite (α-Fe_2_O_3_). We find that upon ionization the hole relaxes from a delocalized band state to a polaron localized on a single iron atom with localization induced by tetragonal distortion of the six surrounding iron–oxygen bonds. This distortion is responsible for sluggish hopping transport in the Fe-bilayer, characterized by an activation energy of 70 meV and a hole mobility of 0.031 cm^2^/(V s). By contrast, the excess electron induces a smaller distortion of the iron–oxygen bonds resulting in delocalization over two neighboring Fe units. We find that 2-site delocalization is advantageous for charge transport due to the larger spatial displacements per transfer step. As a result, the electron mobility is predicted to be a factor of 3 higher than the hole mobility, 0.098 cm^2^/(V s), in qualitative agreement with experimental observations. This work provides new fundamental insight into charge carrier transport in hematite with implications for its photocatalytic activity.

## 1 Introduction

Understanding charge transport in transition metal oxides is essential to advancing technical frontiers across diverse fields ranging from biogeochemistry, to renewable energy materials and microelectronics. Hematite is a prominent example. It is a naturally abundant n-type semiconductor^1^ and plays a crucial role in redox cycling,^2,3^ bioremediation,^4^ and colloid chemistry.^5^ Moreover, the mineral oxide has a visible spectrum band gap and consequently has attracted much interest as a photoanode material for water splitting,^6−11^ although problems remain including low mobility and short carrier lifetimes.^12^

Given the large number of studies that this material has inspired over the past decades, it is noteworthy that the intrinsic electron and hole mobilities of undoped hematite remain experimentally poorly constrained. At the same time, ever improved computational methods are at our disposal to investigate charge transport. In particular, critical advances in the approximations used in density functional theory (DFT) enable ever increasing accuracy in describing the underlying physics controlling charge transport in this material.

At most practical temperatures, charge transport in hematite occurs through thermally activated hopping of polarons, localized lattice distortions that lower the energy of the excess electron or hole such that it becomes self-trapped.^13^ As hematite is a native n-type semiconductor and is frequently further doped with electron donors,^14−16^ the electron polaron has received much greater attention and has been shown with both wave function^17−19^ and density functional theory^15,20,21^ methods to localize on Fe atoms via their 3d states. The nature of the electron hole polaron however appears to be more disputed, where some groups have shown that it localizes on Fe atoms,^18^ others on O atoms via 2p states,^22,23^ and one group even finding that there is no localized hole polaron.^15^

In the absence of conclusive experimental evidence^24,25^ our previous work^26^ sought to clarify this situation using the gap-optimized hybrid functional HSE06,^27^ with large supercells under periodic boundary conditions, removing some of the complications and uncertainties present in earlier calculations. We have demonstrated that the electron hole polaron localizes on a single iron atom, with octahedral distortion of the surrounding iron–oxygen bonds and a change in spin moment of +0.66, consistent with hybrid DFT^23^ and Hartree–Fock^18^ calculations from other groups. The electron polaron however delocalizes equally across two neighboring iron atoms as a consequence of the lower reorganization energy for electrons compared to holes,^26^ with a smaller change in spin moment of +0.23 over each iron atom.

**Figure 1:**
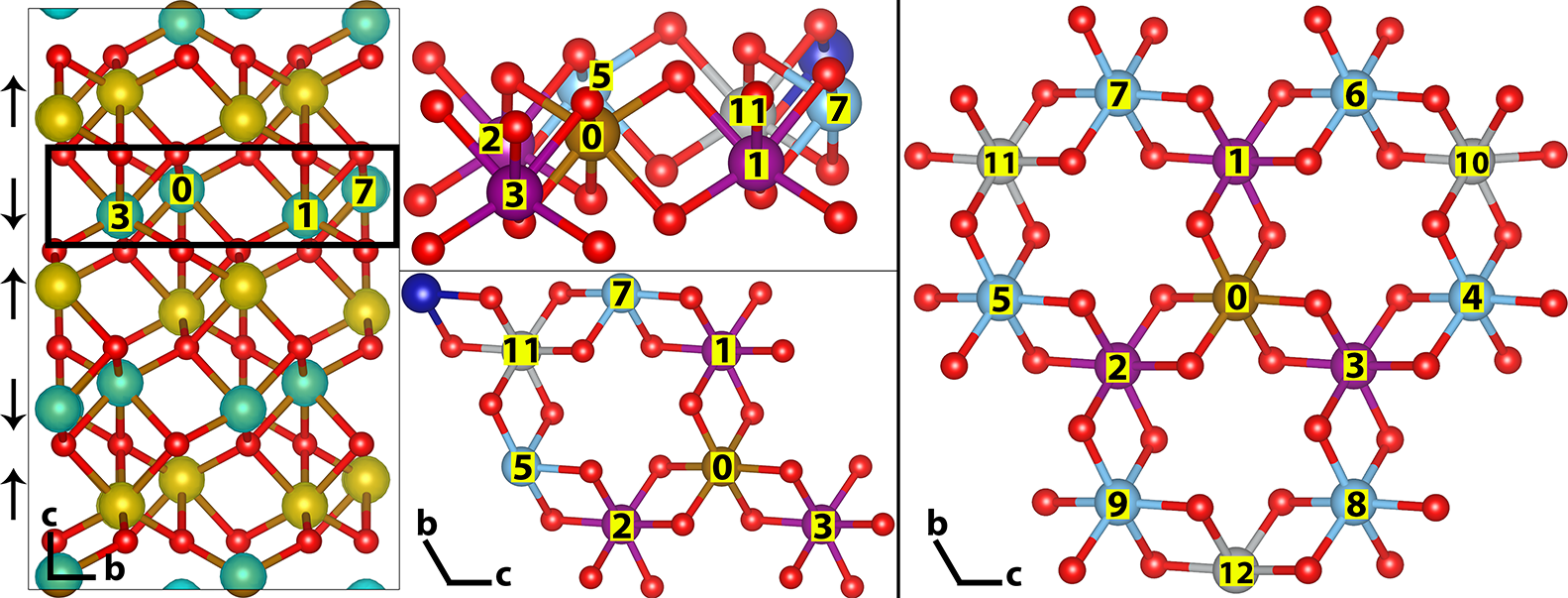
Structure of hematite. 2 ×2 × 1 supercell spin density (left), with a single AFM plane highlighted in black shown color coded by distance from a central iron atom 0 (middle). A single AFM plane truncated to third nearest neighbors is shown (right). There are three first nearest neighbors (purple) at a distance of 2.97 Å, six second nearest neighbors (blue) at 5.04 Å, and three third nearest neighbors (gray) at 5.87 Å. AFM spin orientation is indicated by arrows to the left of the figure.

In this paper we turn our attention to the calculation of electron and electron hole diffusivity and mobility for hematite. In particular, we would like to understand how the 2-site delocalized electron moves along the lattice and how its mobility differs from that of the electron hole. Shown highlighted in Figure 1 is a single antiferromagnetic (AFM) plane of hematite, referred to as an iron bilayer, where both the excess electron and excess hole may localize. We consider the mobility only within this basal plane, as electrical conductivity measurements show that conduction is 4 orders of magnitude greater than in the perpendicular direction.^28,29^

To this end, we calculate the electron transfer parameters and rates for electron and electron hole transfer in hematite using constrained density functional theory (CDFT). CDFT is an established method for generating diabatic electronic states and calculating ET parameters in molecular systems,^30−35^ but applications to condensed phase/periodic systems remain rare to date. In previous related work we used a plane-wave implementation of CDFT to calculate ET parameters and rates for electron tunnelling between F-center defects in MgO.^34^ Though, applications to late (spin density rich) transition metal ions remained computationally prohibitive. In this work we take advantage of a recent and very efficient periodic atomic-orbital implementation of CDFT^36^ to calculate at the hybrid DFT level all the ET parameters required to predict the charge mobility of electrons and holes in bulk hematite. As with our previous work, we stress it is only due to the increasing efficiency of computer codes and platforms that it is possible to perform such expensive hybrid CDFT calculations in combination with large supercells.

We find that the hole polaron in hematite localizes onto a single iron atom with tetragonal distortion of the six surrounding iron–oxygen bonds. The 3-fold degenerate tetragonal distortion of the Fe octahedron is responsible for the low hole mobility in hematite, calculated as 0.031 cm^2^/(V s), a property well recognized to bear directly upon the photocatalytic behavior of hematite.^6,9^ The higher mobility of the electron polaron, 0.098 cm^2^/(V s), is attributed to a delocalization over two neighboring iron atoms, advantageous for charge transport due to the larger spatial displacements per transfer step.

The remainder of the paper is organized as follows. Section 2 presents a breakdown of the required electron transfer theory, with Section 3 detailing the computational methods used including details of the CDFT calculations. Section 4.1 presents the CDFT results for the hole polaron, Section 4.2 for the electron polaron, and Section 4.3 the calculated mobilities. Section 5 presents a discussion of the results, and concluding remarks are made in Section 6.

## 2 Theory

For calculation of the required electron transfer (ET) parameters and mobilities we adopt the same ET theory as used in previous studies of hematite,^18^ and in other CDFT calculations.^34^ The semiclassical expression for the rate of ET in a donor–acceptor complex derived from transition state theory in the harmonic approximation and Landau–Zener theory has the form^37,38^
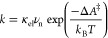
1with the electronic transmission coefficient κ_el_ = 2*P*_LZ_/(1 + *P*_LZ_) where *P*_LZ_ = 1 – exp(−2*πγ*) is the Landau–Zener transition probability with γ as the adiabaticity parameter defined as . ⟨|*H*_ab_|^2^⟩_TS_ is the squared electronic coupling averaged over the transition state (TS) configurations, ν_n_ is the effective nuclear frequency along the reaction coordinate, *ΔA*^‡^ is the activation free energy, λ is the reorganization free energy, *k*_B_ is the Boltzmann constant, and *T* is the temperature.^39^ For an effective nuclear frequency we use the same value as that used by Rosso and co-workers,^18^ the energy of the highest infrared active longitudinal optical mode phonon 1.85 × 10^13^ s^–1^. We note this is very close to the experimental Fe–O stretch vibration 1.72 × 10^13^ s^–1^.^40^

The general expression for the activation free energy *ΔA*^‡^ valid in the nonadiabatic, adiabatic, and intermediate regimes is^41^
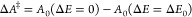
2
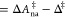
3where *A*_0_ is the free energy curve for the adiabatic electronic ground state for electron transfer taking the vertical energy gap, *ΔE*, as reaction coordinate
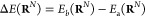
4*E*_*a*_ and *E*_*b*_ are the electronic energies for initial and final diabatic states *a* and *b*, **R**^*N*^ is the nuclear configuration, *ΔE* = *ΔE*_0_ is the position of the minimum of state *a*, and *ΔE* = 0 is the position of the TS.

*ΔA*_na_^‡^ is the activation free energy on the diabatic electronic states
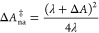
5and Δ^‡^ is a correction that becomes important when the electronic coupling *H*_ab_ is large (|*H*_ab_| > ∼0.01–0.1 λ)
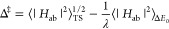
6with the assumption that the free energy difference, *ΔA*, between the initial and final state is zero, which is the case in hematite due to symmetry. By ignoring thermal effects of phonons on electronic coupling and reorganization free energy, the activation free energy is approximated by the activation energy, *ΔE*^‡^,
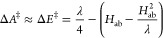
7where *H*_ab_ is taken at the TS and the reorganization energy λ is calculated as
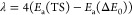
8where *E*_a_(TS) and *E*_a_(*ΔE*_0_) are the electronic energies of the initial diabatic state at the transition state and minimum energy nuclear configurations, respectively, calculated using CDFT. Note that, for the current system, *E*_a_(*ΔE*_0_) is virtually identical with the DFT (adiabatic) ground state energy at the minimum energy nuclear configuration.

Charge transport in hematite can be modeled as a succession of hops between sites, with associated rate constants calculated from eq 1. The corresponding charge mobility is obtained from the Einstein relation
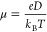
9

Calculation of the diffusion coefficient *D* can be performed through methods such as kinetic Monte Carlo,^42^ or by solving a chemical master equation to obtain the time-dependent charge population of each site as by Giannini et al.^43^ The mean squared displacement (MSD) is calculated from the time evolution of the charge population, and following an initial nonlinear equilibration period the diffusion coefficient is given by the gradient of the MSD^44^
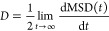
10

As a result of the lattice symmetry, diffusion is isotropic within the basal plane and therefore the calculated mobility tensor becomes a single value.

Alternatively, the diffusion coefficient can be calculated analytically for a 1D chain model as
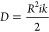
11for the transfer distance *R*, site multiplicity *i*, and rate constant *k*. Rosso and co-workers^17,18^ directly used eq 11 for a 1D model of the 2D basal plane of hematite, with the site multiplicity *i* = 3 to approximately account for the 3 first nearest neighbors of an iron atom in the 2D plane. Adelstein et al.^20^ also used eq 11 for an approximation of the 2D plane, but with *i* = 0.5. Our approach moves beyond these approximations, calculating the full mobility tensor in the basal plane.

## 3 Computational Methods

We use the range-separated hybrid functional HSE06,^27^ with the percentage of exact Hartree–Fock exchange (HFX) modified to 12% to reproduce the experimental band gap of hematite.^45,46^ In previous work we demonstrated that the standard definition of HSE06 with 25% HFX overestimates the experimental band gap of 2.2 eV as 3.6 eV,^47^ and does not satisfy the generalized Koopmans condition.^26^

Initial coordinates were taken from the experimental crystal structure for hematite,^48^ with geometry optimization converged until the residual forces were smaller than 0.02 eV/Å. For a more complete discussion of the structure and setup of bulk hematite refer to ref (26). The only change in this work was that the planewave cutoff was tightened from 400 to 600 Ry to aid in the verification of degenerate structures.

We find that the Hirshfeld spin moment on the Fe atom is a suitable descriptor for the polaronic states. The spin moment changes from −3.95 to −3.29 for the electron hole and −3.95 to −3.72 for each of the two Fe atoms over which the electron polaron is delocalized. Interestingly, the change in charge is not found to be a useful descriptor due to (paired) electron rearrangement, as also noted by other groups.^20,23^

For electron hole transfer between two Fe atoms, Fe_*A*_ and Fe_*B*_, we define the initial (final) ET state as the spin constrained CDFT state with the spin moment on Fe_*A*_ (Fe_*B*_) constrained to −3.29. For electron transfer between two 2-site delocalized Fe pairs, (Fe_1_–Fe_2_)_*A*_ and (Fe_3_–Fe_4_)_*B*_, we define the initial (final) ET state as the spin constrained CDFT state with the spin moment on each of the two iron atoms Fe_1_ and Fe_2_ (Fe_3_ and Fe_4_) constrained to −3.72. These constraints ensure that for any geometry (including transition state geometry) diabatic states are obtained that resemble very closely the DFT electronic ground state of the electron hole or electron polaron in the global minimum energy structure.

Other definitions of the spin constraint would be possible. For instance, one could include the first shell oxygen atoms but we found that their spin moment is rather small and their inclusion in the constraint is not beneficial. Moreover, one could constrain the spin density difference between donor and acceptor groups which is a common choice in CDFT calculations.^34^ However, we found that a single absolute spin constraint on the Fe atoms in question is the most suitable choice in the present case.

To setup the CDFT calculations, first the polaron is localized on each of the desired iron atoms, typically by offsetting the local Fe–O bond lengths to facilitate polaron formation at this location. After geometry optimization to form the charged DFT ground state, linear interpolation is performed to create the transition state geometries. These transition state geometries are then used to calculate both the electronic couplings and reorganization energies (eq 8) using CDFT. This is performed by constraining the spin moment of the iron atoms to the spin moment of the charged ground state given above, thus generating the diabatic electronic states at the transition state geometry.

## 4 Results

### 4.1 Hole Polaron

**Figure 2:**
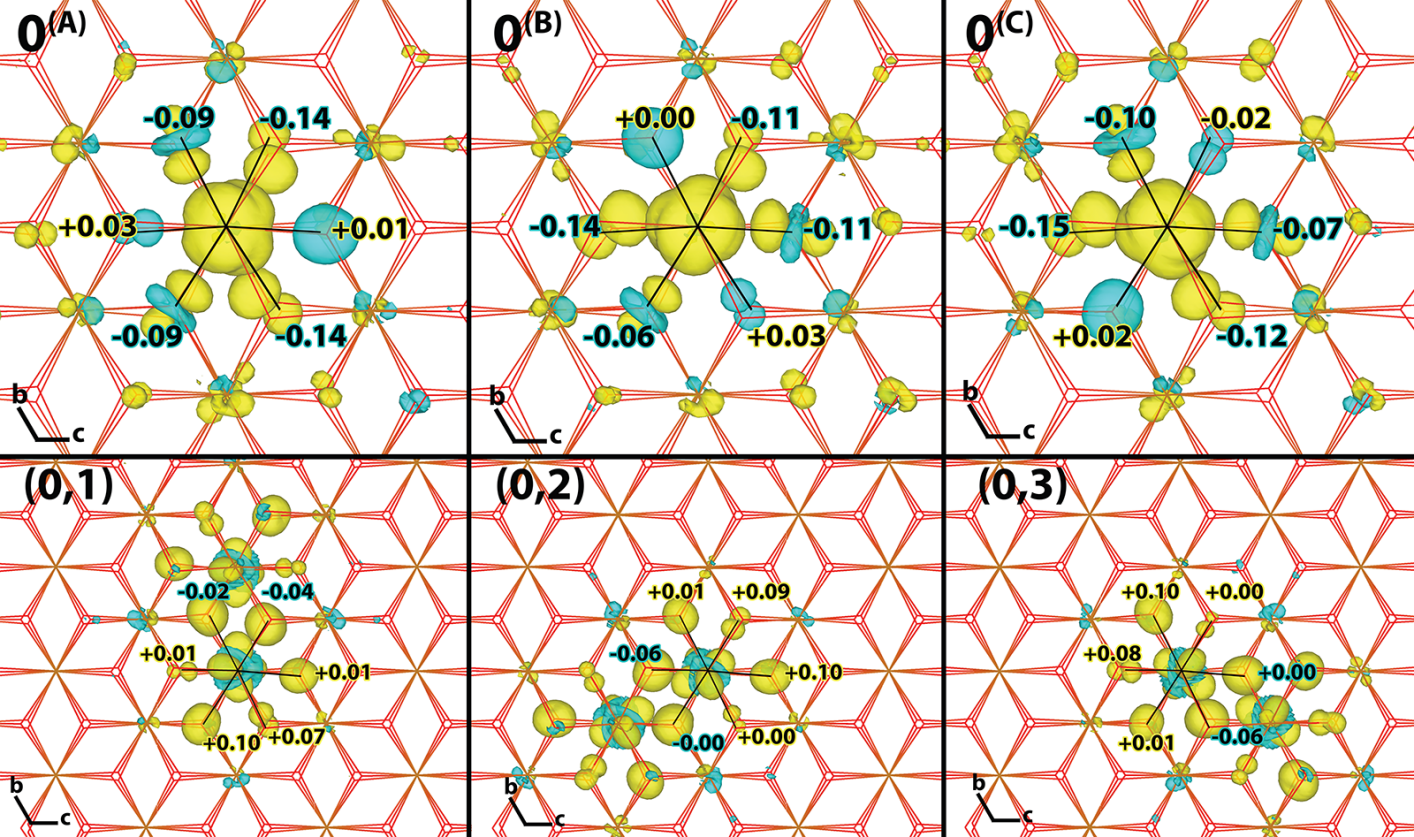
Excess hole and excess electron in hematite. 4 × 4 × 1 supercell excess spin density for ground state hole polaron (top) and electron polaron (bottom), from DFT calculations. A hole polaron localized on an iron atom has three degenerate structures (A, B, and C) due to the octahedral distortion of the Fe–O bonds. As the electron polaron is localized across two iron atoms, for any combination of first nearest neighbors , the structures are also degenerate. Bond length differences with respect to neutral hematite between the iron atom and bonded oxygen atoms are shown in Angstrom.

The electron hole polaron, shown in Figure 2 (upper row), is mainly localized on a single Fe atom and to a lesser extent on first shell oxygen atoms. It is stabilized by an octahedral distortion of the iron–oxygen bonds. There is a contraction of four equatorial Fe–O bonds, and a very slight expansion of two axial Fe–O bonds. These changes in bond lengths are in response to the removal of electron density in the equatorial plane, more specifically in response to removal of an electron from a d_*x*^2^–*y*^2^_ orbital. Similar tetragonal elongation is observed in the Jahn–Teller effect of high spin d^4^ complexes; however, this is not strictly Jahn–Teller distortion, as there are two distinct groups of Fe–O bond lengths of 1.94 and 2.12 Å in the neutral geometry due to the iron bilayer.^48^

Importantly, we find that, in the hematite lattice, the tetragonally distorted structure can be realized in three equivalent ways giving exactly the same electronic energy; see Figure 2 (upper row). Each of these symmetry-related structures can be transformed into one another by lattice vibrations. To the best of our knowledge this 3-fold degeneracy of the hole polaron has not been previously explored, and its effect on the mobility has not been investigated.

Due to the 3-fold structural degeneracy, there are 3 × 3 = 9 possible transition state structures for hole transfer between an Fe atom and any of the three nearest neighbors. However, only 5 of these 9 combinations are unique featuring different donor–acceptor orbital combination and electronic coupling (see Table 1) and reorganization and activation energy (Table 2). Note that the same set of five unique electronic couplings exist for all three nearest neighbors.

**Table 1:** First Nearest Neighbour Electronic Coupling for the Hole Polaron in Bulk Hematite, Accounting for All Possible Degenerate Structures of Atoms 0 and 1^a^

Electronic coupling /meV			
	1^(*A*)^	1^(*B*)^	1^(*C*)^
0^(*A*)^	203	110	101
0^(*B*)^	110	53	39
0^(*C*)^	101	39	53

a All other atom combinations can be inferred by symmetry; e.g., highest coupling direction 0^(*A*)^1^(*A*)^ is equivalent to 0^(*B*)^2^(*B*)^.

**Table 2:** Summary of Parameters and Rates for Hole and Electron Rransfer^a^

**Hole polaron**						
*r* (Å)	Neighbor	*H*_ab_ (meV)	λ (meV)	*ΔE*^‡^ (meV)	κ_el_	*k* (s^–1^)
2.97	0^(*A*)^-1^(*A*)^	203	652	23	1.0	7.5 × 10^12^
	0^(*A*)^-1^(*B*)^	110	814	108	1.0	2.8 × 10^11^
	0^(*A*)^-1^(*C*)^	101	784	108	1.0	2.8 × 10^11^
	0^(*B*)^-1^(*B*)^	53	865	166	0.9	2.5 × 10^10^
	0^(*B*)^-1^(*C*)^	39	881	183	0.7	1.1 × 10^10^
						
	Average	147^b^	752^c^	70	1.0	1.2 × 10^12^
						
5.04	0^(*A*)^-4^(*A*)^	15	1050	248	0.2	2.2 × 10^8^
	0^(*A*)^-5^(*A*)^	8	1050	255	0.1	5.3 × 10^7^
	0^(*A*)^-6^(*A*)^	15	1028	242	0.2	2.8 × 10^8^
	0^(*A*)^-7^(*A*)^	30	1016	225	0.5	1.5 × 10^9^
	0^(*A*)^-8^(*A*)^	28	1022	228	0.5	1.2 × 10^9^
	0^(*A*)^-9^(*A*)^	16	1034	243	0.2	3.0 × 10^8^
						
	Average	21^b^	1032^c^	237	0.3	5.7 × 10^8^
						
5.87	0^(*A*)^-10^(*A*)^	3	1087	269	0.0	4.4 × 10^6^
	0^(*A*)^-11^(*A*)^	9	1106	268	0.1	3.9 × 10^7^
	0^(*A*)^-12^(*A*)^	45	1026	213	0.8	3.6 × 10^9^
						
	Average	32^b^	1061^c^	234	0.5	1.1 × 10^9^

**Electron polaron**						
2.97	(0,1)-(0,2)	26	363	67	0.6	8.0 × 10^11^
						
5.04	(0,2)-(1,6)	57	522	80	0.9	7.7 × 10^11^
	(0,2)-(1,7)	19	572	125	0.3	4.9 × 10^10^
	(0,1)-(5,11)	10	641	150	0.1	5.7 × 10^9^

a Electronic coupling, reorganization energy, activation energy, transmission coefficient, and rate constant for bulk hematite at room temperature. For the hole polaron the Boltzmann average for the electronic coupling and reorganisation energy is calculated for each nearest neighbour, while for the electron polaron there is only a single second nearest neighbour with an adiabaticity parameter greater than one and therefore this is not necessary.

b Boltzmann average for electronic coupling *H*_ab_ = (∑_*i*_*H*_ab,i_^2^*e*^–λ_*i*_/4*k*_*B*_*T*^/∑_*i*_*e*^–λ_*i*_/4*k*_*B*_*T*^)^1/2^ for hole transition states i; see SI for further detail.

c Boltzmann average for reorganization energy  for hole transition states i; see SI for further detail.

**Figure 3:**
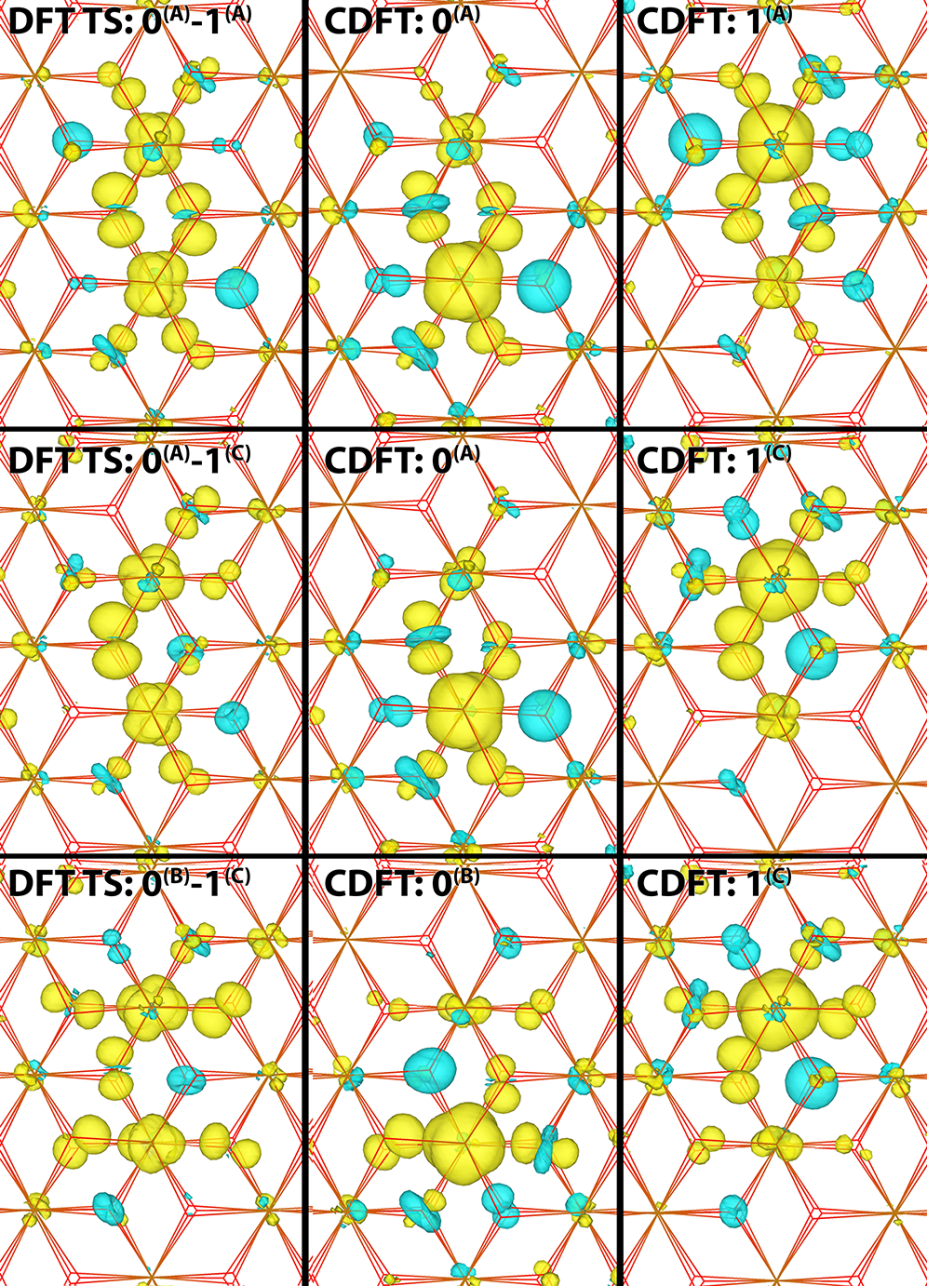
Electron hole at the transition state geometry. 4 × 4 × 1 supercell excess spin density for electronic ground state, obtained from DFT calculations (left column) and for the diabatic initial (middle column) and final hole transfer states (right column). The diabatic states are obtained from CDFT with a spin constraint on the Fe atom 0 or 1 respectively, see Figure 1 for atom labelling. Only three of the five unique transition state geometries for nearest neighbour hole transfer are shown, see Table 2 for hole transfer parameters.

The five unique nearest neighbor couplings can be placed into three groups shown in Figure 3: highest coupling (203 meV) where the polaron in initial and final ET states has d_*x*^2^–*y*^2^_ orbitals aligned along the Fe–Fe direction shown in Figure 3 (upper row); moderate coupling (101, 110 meV) where in one polaronic state the d_*x*^2^–*y*^2^_ orbital is aligned along the Fe–Fe direction shown in Figure 3 (middle row); and low couplings (39, 53 meV) where in neither polaronic state the d_*x*^2^–*y*^2^_ orbital is aligned along the Fe–Fe direction shown in Figure 3 (lower row). The reorganization energies of the five unique combinations are also slightly different; all ET parameters and rates are summarized in Table 2.

Similar considerations apply for second nearest neighbors and beyond. However, as accounting for every structural combination of the hole polaron for all of the second and third nearest neighbors would become too computationally demanding, we choose to only consider these for the (A) orientation of the hole polaron. As Table 2 shows, the decay of the electronic coupling with distance and the increase in reorganization energy is such that the interaction of the hole polaron with its second and third nearest neighbors is negligible. This means that only nearest neighbor charge transfer processes are important for the hole polaron in bulk hematite, consistent with work from other groups.^17,18^

### 4.2 Electron Polaron

In previous work we found that the excess electron in hematite is delocalized over two neighboring iron sites in the DFT electronic ground state.^26^ According to electron transfer theory, this suggests that electronic coupling between 1-site localized excess electronic states is so large that they are are no longer stable states, that is, they no longer correspond to a minimum of the ground state potential energy surface. This is the case as soon as *H*_ab_ > λ/2.^41^ We are now in a position to further verify this hypothesis using CDFT.

Indeed, using CDFT to constrain the excess electron on a single Fe atom, we obtain a very large coupling value of 407 meV (4 × 4 × 1 supercell), while an upper limit for reorganizations energy for nearest neighbor hopping of the 1-site localized electron polaron can be estimated to be 0.49 eV;^26^ hence, *H*_ab_ > λ/2. Thus, both DFT calculation of the electronic ground state (adiabatic representation) and CDFT calculations of spin-localized states (diabatic representation) suggest that the 1-site localized electron polaron is unstable and delocalizes over two adjacent sites.

**Figure 4:**
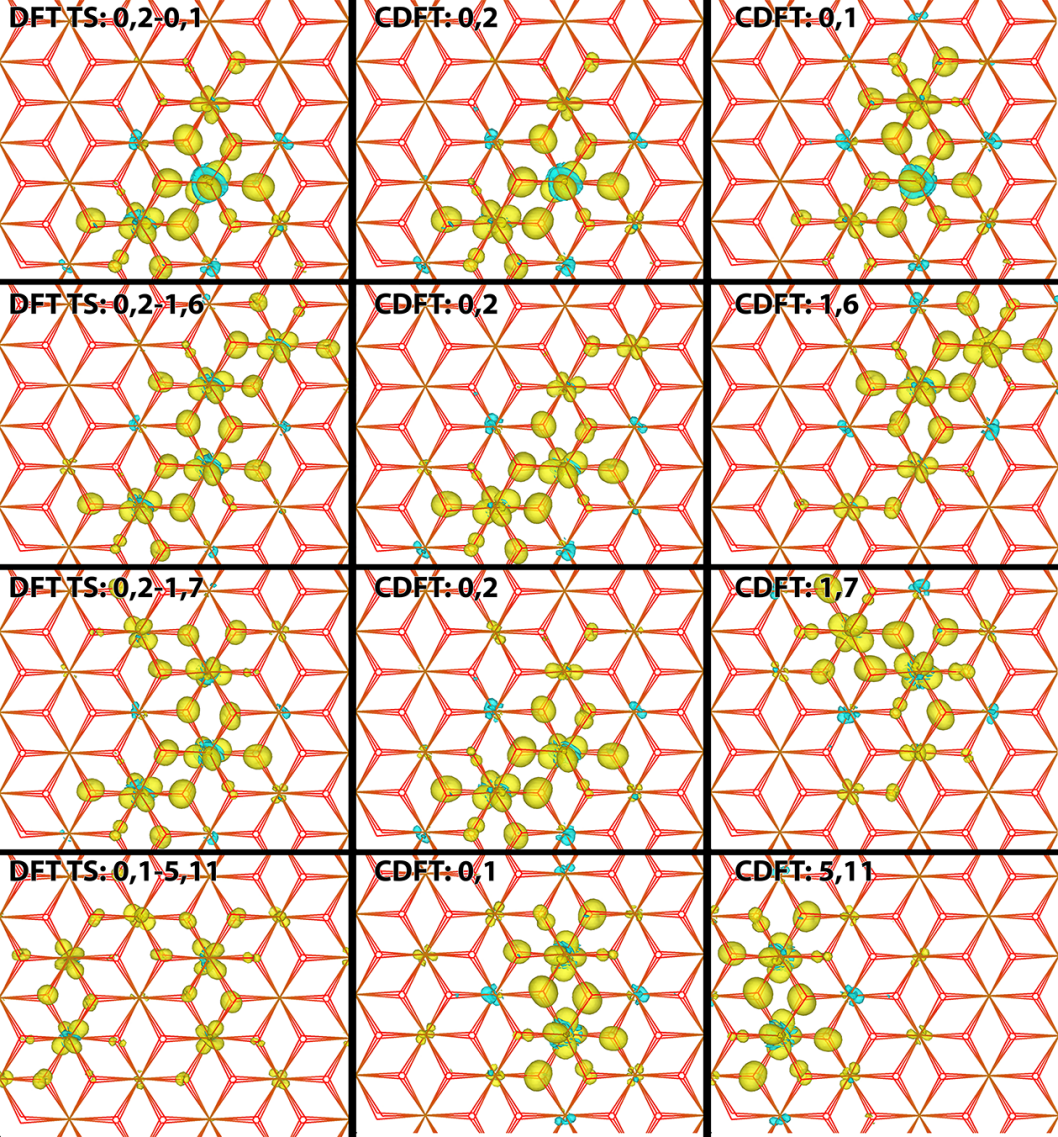
Excess electron at the transition state geometry. 4 × 4 × 1 supercell excess spin density for electronic ground state, obtained from DFT calculations (left column) and for the diabatic initial (middle column) and final electron transfer states (right column). The diabatic states are obtained from CDFT with a spin constraint on the two Fe atoms as indicated, see Figure 1 for atom labeling. The first row is for electron transfer over the shortest distance (2.97 Angstroms) and the second to fourth row for electron transfer over the second shortest distance (5.04 Angstroms). See Table 2 for electron transfer parameters.

Considering a given iron atom “0”, delocalization can occur over one of the three first nearest neighbors of 0: (0,1); (0,2); or (0,3) (see Figure 2 for numbering scheme). These states are energy degenerate due to the symmetry of the lattice. There are several possible charge transfer events of 2-site delocalized electron polaron. The shortest transfer (2.97 Å between centers of excess spin) includes the transitions: (0,1)-(0,2); (0,1)-(0,3); and (0,2)-(0,3) where (0,1)-(0,2) denotes the electron transfer from the 2-site delocalized state (0,1) to the 2-site delocalized state (0,2), and similarly for the other electron transfers. All of these three electron transfers are equivalent by symmetry. The coupling between these adjacent states is surprisingly small given that they share an Fe atom with significant excess spin density in both states. The reason is that the Fe t_2g_ orbital carrying the excess spin density is rotated by 120° with respect to one another in the two diabatic electronic states; see Figure 4 (upper row). This results in a small overlap of the two (nonorthogonal) diabatic CDFT electronic wave functions and thus a small electronic coupling.

Transfers over the next largest distances (5.04 Å between the centers of excess spin) includes the transitions: (0,2)-(1,6); (0,2)-(1,7); and (0,1)-(5,11) as shown in Figure 4. The highest coupling is found for combination (0,2)-(1,6) where the iron t_2g_ orbitals which the excess electron occupies are orientated parallel. While the combination (0,1)-(5,11) has the same center of mass distance, the iron atoms do not share Fe–O bonds as for the other two transition states, and as such the coupling is the lowest of the three.

### 4.3 Electron Hole and Electron Mobilities

The three structurally degenerate states of the hole polaron are expected to interconvert fast, on the time scale of the high frequency lattice modes (∼10^13^ s^–1^). This allows us to perform a Boltzmann average over the electronic couplings and reorganization energy for the nine possible transitions *i*, with weights proportional to exp(−*βλ*_*i*_/4), and use the averages for calculation of a nearest neighbor hopping rate (eq 1). The latter is ∼10^12^, slow enough to support degenerate mixing of hole states by phonons. The rates for second and third nearest neighbor hops are orders of magnitude smaller showing that only first nearest neighbor hops are important for hole polaron transport.

Table 3 gives the hole mobility for bulk hematite in the 2D (Fe bilayer) plane at room temperature, calculated by solving a chemical master equation to get the MSD and diffusion coefficient (eq 10) as by Giannini et al.^43^ Inclusion of the six second nearest neighbors and three third nearest neighbors of the hole polaron increases the mobility only from 3.08 × 10^–2^ to 3.10 × 10^–2^ cm^2^/(V s). To the best of our knowledge this is the first calculation of a mobility tensor in hematite, which fully accounts for the effect of the 2D conduction environment.

**Table 3:** Summary of Results and Comparison with Literature^a^

**Hole polaron**							
Source	Dopant	*H*_ab_ (meV)	λ (meV)	*ΔA*^‡^ (meV)	*T* (K)	μ_lit_ (cm^2^/(V s))	μ_2D_ (cm^2^/(V s))
This work		147	752	70	300		3.1 × 10^–2^
This work		147	752	70	1000		6.1 × 10^–2^
Cluster HF^18^		200	1590	220	298	1.7 × 10^–4^	8.3 × 10^–5^
Estimate^50^				100	300	1 × 10^–2^	
Experiment^51^	Mg			200	1000	9.1 × 10^–1^^b^	

**Electron polaron**							
This work		57	522	80	300		9.8 × 10^–2^
This work		57	522	80	1000		2.0 × 10^–1^
Cluster HF^17^		200	1200	110	298	6.2 × 10^–2^	2.6 × 10^–3^
Cluster HF^18^		190	1420	190	298	5.6 × 10^–4^	2.9 × 10^–4^
Bulk DFT^20^		41	(674)^c^	130	300	9.0 × 10^–3^	1.4 × 10^–3^
Bulk DFT^21^		40	800	(162)^c^	300		6.2 × 10^–4^
Estimate^50^				100	300	1 × 10^–2^	
Experiment^52^	Nb, Zr				960–1500	1 × 10^–1^	
Experiment^51^	Ti			170	1000	2.8 × 10^–1^^b^	
Experiment^53^	2%Ti			80	780	2 × 10^–2^	
Experiment^54^	3%Ti			118	290	4 × 10^–2^	
Experiment^54^	5%Ti			116	290	4.6 × 10^–1^	
Experiment^54^	9%Ti			116	290	1.4 × 10^–1^	

a To facilitate direct comparison, both the mobility cited in the paper (μ_lit_) as well as mobilities recalculated using eqs 1–9 for the 2D plane (μ_2D_) are provided. Literature results are presented in chronological ordering by method

b Calculated from fitted mobility equation given in paper, valid above 923 K.

c Calculated in this work from values in paper.

We have also examined finite size effects for the hole polaron, finding that the smaller 2 × 2 × 1 supercell commonly used in the literature provides both an underestimate of the electronic couplings and an overestimate of the reorganization energy and therefore a lower mobility. This is attributed to the smaller reorganization of the first coordination shell (“inner sphere”) in the larger supercell (see SI for more details).

For the electron polaron, we consider both the three transitions over the shortest possible distance and the transition over the next largest distance having the highest electronic coupling. Due to the 2-site delocalized electron polaron structure, there are only four symmetry related second nearest neighbors to which the polaron may hop (see SI Figure 5). This introduces a similar complication to the hole polaron, that for a single energy degenerate structure of the electron polaron the mobility is locally anisotropic. As there are three energy degenerate structures for a given electron polaron, the overall mobility remains isotropic. The electron mobility calculated for hopping to first nearest neighbors is 2.0 × 10^–2^ cm^2^/(V s) while for second nearest neighbors it is 7.8 × 10^–2^ cm^2^/(V s), as a consequence of how the coupling for first nearest neighbors is smaller than for second nearest neighbors due to unfavorable orientation of orbitals. Combining both first and second nearest neighbor mobility gives a total mobility of 9.8 × 10^–2^ cm^2^/(V s). Hops across larger distances are not expected to contribute to electron mobility, as the electronic coupling for those decays very quickly.

## 5 Discussion

**Figure 5:**
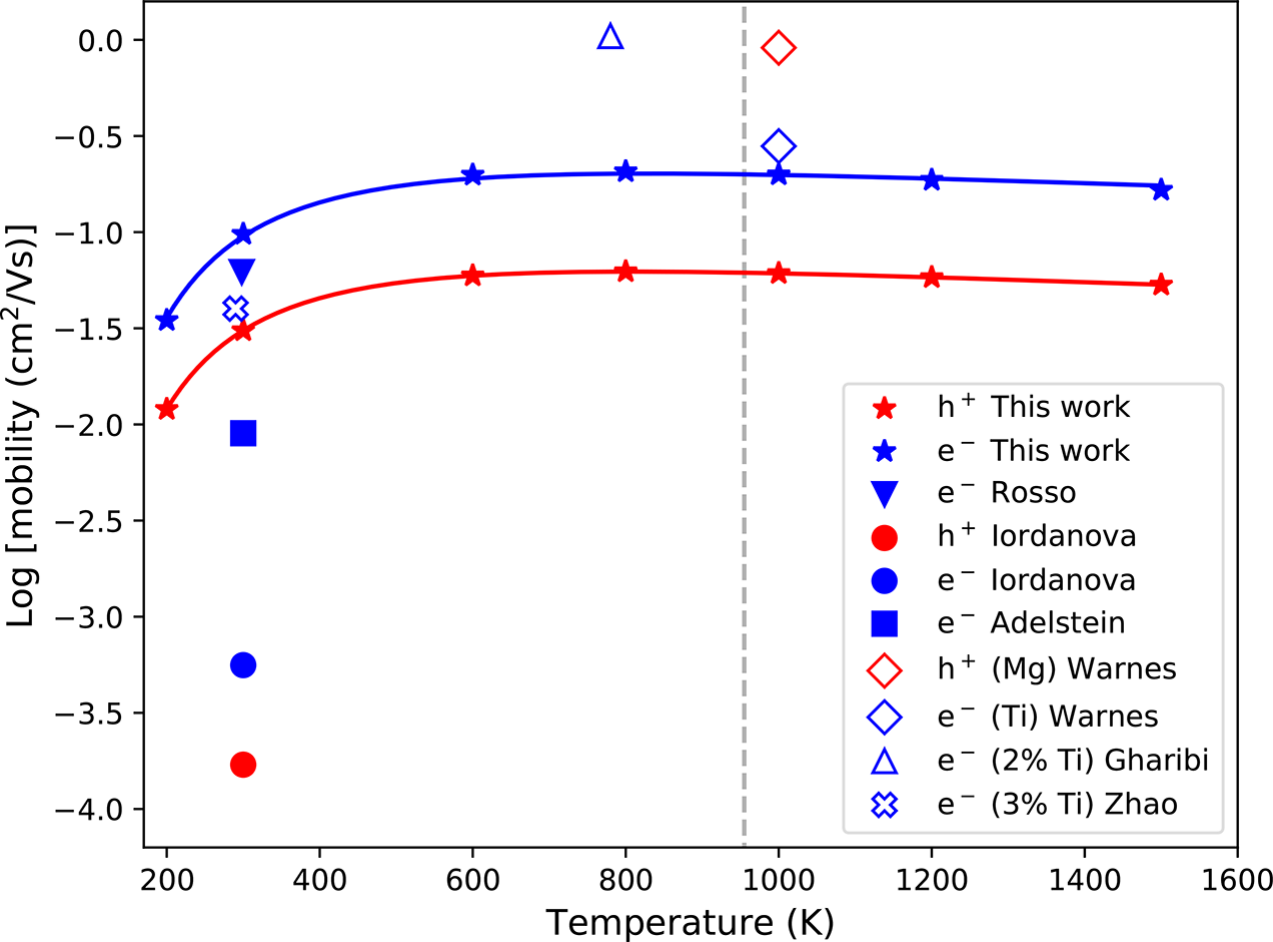
Electron and hole mobilities as a function of temperature. Literature calculated mobilities indicted with solid filled markers, doped experimental mobilities as unfilled markers. Our results are valid below the Neel temperature (*T* = 955 K),^49^ indicated with a dashed line. See SI for an alternate plot where literature calculated mobilities are recalculated using eqs 1–9.

Table 3 shows a comparison of our calculated and literature results, with Figure 5 showing this comparison plotted as mobility against temperature. A direct comparison of different mobility calculations is difficult due to the different methods used, and therefore we alleviate this somewhat by comparing to mobilities obtained according to eqs 1–9 using the reported literature values for electronic couplings and reorganization energy.

Rosso and co-workers^17,18^ performed Hartree–Fock calculations of both hole and electron mobility for small hematite clusters, considering up to second nearest neighbors. Both their couplings and reorganization energies tend to be considerably higher resulting in larger activation energies and lower mobilities for both electrons and holes. Though, one early estimate reported by Rosso and co-workers^17^ for electron mobility is within a factor of 1.6 of our current best estimate. In previous work^26^ we attributed the larger reorganization energy of cluster models to the absence of lattice strain effects present in the bulk structure, as well as the use of Hartree–Fock calculation which tends to overbind excess charge.

Adelstein et al.^20^ and Behara et al.^21^ calculated electron mobilities using a similar periodic supercell approach as in this work but with DFT+U in place of hybrid CDFT to model the polaronic states. Their reorganization energies are similar, albeit slightly higher than ours and significantly smaller than typical values for cluster models. In fact, Behara reported values for bulk hematite around half of that for the 1D chain.^21^ Their electronic coupling values are also very similar to our estimates, but this is a coincidence, as our electronic polaronic states are 2-site delocalized whereas theirs are localized on a single iron atom. The 2-site delocalization also permits larger transfer distances for a single hop resulting in higher mobilities than the 1-site localized polaron (see *R*^2^ dependence, eq 11). This is partly the reason for the higher electron mobilities that we obtain in this current work compared to Adelstein et al. and Behara et al. Interestingly, Adelstein et al. in their calculations did find that there was some degree of delocalization of the electron polaron over two iron atoms, identified from both a shorter Fe–Fe bond length and from the magnetic moment.

While other groups have attempted a justification of their results via comparison to experiment, this is problematic as there are no experimental results for the mobility of pure (undoped) hematite. The available experimental mobilities are all for doped hematite, sometimes for temperatures above the Neel temperature where hematite is no longer antiferromagnetic (955 K).^49^ Further, as there are no direct measurements of either the reorganization energy or couplings there are multiple combinations of each that would compare well with the observed mobilities.

The most suitable experimental data for comparison are probably the ones reported by Zhao et al.^54^ for electron mobility in 3% and 5% Ti-doped hematite. These values are within a factor of 2.5 of our computed mobilities for pure hematite, which is reassuring despite the above-mentioned caveats.

## 6 Conclusion

In this work both the electron and hole mobility has been calculated for hematite using spin-constrained gap-optimized hybrid density functional theory with large supercells. Where previous studies have only considered coupling between a single nearest neighbor or a single orientation of the polaron, we account for all possible degenerate polaron structures and coupling with up to third nearest neighbors. In addition, for the first time the mobility is calculated for the full 2D Fe bilayer rather than for a 1D model.

The CDFT calculations reported herein provide further evidence that the excess electron is delocalized over two iron sites and hops across the hematite crystal as a two-site delocalized polaron. While the activation energy for these hops is slightly higher, the hopping distance is larger than for the 1-site localized hole polaron. As a consequence, the electron mobility is predicted to be a factor of 3 higher than the hole mobility.

Charge transport has been identified as a key issue for the use of hematite in a number of technological applications, especially in photocatalysis and photoelectrochemistry.^6,9,11,12^ Our study provides a comprehensive and detailed understanding of the physical mechanisms that lead to the sluggish transport of charge carriers in bulk hematite. This sets the scene for similar calculations at the hematite/liquid water interface, for which we have recently carried out *ab initio* molecular dynamics simulations.^55,56^ In particular, it will be important to understand if and how the presence of water at the interface changes the picture obtained for the bulk material and how this depends on the specific surface cut under investigation and the protonation state of the surface.^57^ Such investigations, which the current work has now made possible, could help refine models and resolve ongoing questions, about rate-limiting transport processes governing photocatalytic water splitting efficiency at hematite/water interfaces.^11,12^ Work toward this goal is currently being carried out in our laboratory.

We have shown in this work that CDFT is a useful tool for the prediction of charge mobilities in an ubiquitous oxide material. The method is generally applicable to semiconducting materials and may be applied to other oxides of technological interest for the study of intrinsic charge transfer processes or for charge transfer between defects. Moreover, the CDFT approach is well suited for the study of interfacial charge transfer processes between different semiconductors or between semiconductor electrodes and liquids. It could thus become an essential tool for the emerging field of *ab initio* electrochemistry.

## Supporting Information Available

Finite size effects of electronic coupling and reorganization energy, electronic coupling from alternative methods, details of Boltzmann average of electronic coupling and reorganization energy, images of electron and hole polaron, schematic describing polaron hopping scheme, mobility with recalculated literature values, correlation between overlap and electronic coupling, details of CDFT code (PDF)
