# Supplementary material for: Electron and Hole Mobilities in Bulk Hematite from Spin-Constrained Density Functional Theory
Source: J Am Chem Soc. 2022 Mar 3;144(10):4623–32. doi: 10.1021/jacs.1c13507 (PMC9097473; doi:10.1021/jacs.1c13507)
Supplement: Supplementary file 1 — ja1c13507_si_001.pdf [file ja1c13507_si_001.pdf]

Supplementary Information: Electron and hole mobilities in bulk hematite from spin-constrained density functional theory

Christian S. Ahart, Kevin M. Rosso and Jochen Blumberger

Supplementary Information

Finite size effects

Table 1: Comparison of first nearest neighbour electronic coupling and reorganisation energy for the hole polaron, 2x2x1 and 4x4x1 supercells.

	Supercell size			
	2x2x1	4x4x1	2x2x1	4x4x1
Neighbour	H_{ab} / meV		λ / meV	
$0^{(A)}$-$1^{(A)}$	167	203	843	652
$0^{(A)}$-$1^{(B)}$	36	39	1012	881
$0^{(A)}$-$1^{(C)}$	51	53	1035	865

The results shown in this work are for the 4x4x1 supercell of hematite, with a total of 480 atoms and 4800 electrons. It could be argued that the 2x2x1 supercell (120 atoms, 1200 electrons) is large enough for the first nearest neighbour interactions. Table 1 shows that this is true for the weak coupling directions, but not for the highest coupling direction for which we observe a non-negligible increase from 167 to 203 meV. We analysed the origin for this by preparing a transition state geometry in the 2x2x1 supercell that has exactly the same geometry as the one obtained for the 4x4x1 supercell. The coupling is virtually identical in both cases which means that the increase in coupling going from the 2x2x1 to the 4x4x1 supercell is due to a slightly different geometry of the transition state structure in the 4x4x1 supercell (rather than due to other finite size effects such as, e.g., polarisation effects due to polaron images). The slight differences in nuclear relaxation are also indicated by a small decrease in reorganisation energy going from 2x2x1 to 4x4x1, which we attribute to a smaller reorganisation of the first coordination shell ("inner sphere") in the larger supercell.

Use of a 4x4x1 supercell is essential for the calculation of ET parameters for third nearest neighbours for the hole and second nearest neighbour for the electron polaron. This supercell should be large enough as the distance to the third nearest neighbour of the hole polaron is a factor of 3 smaller than to the distance to the closest periodic image. Remaining finite size effects could not be investigated as supercells larger than 4x4x1 are computationally unfeasible at the moment.

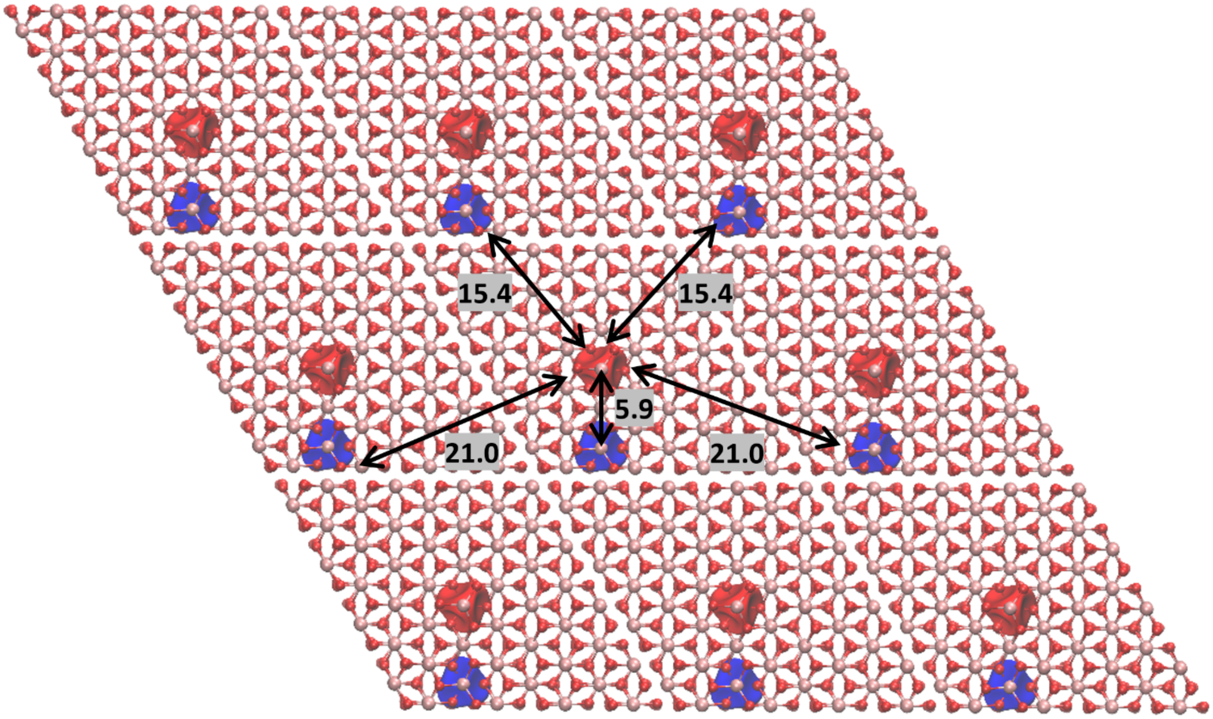

Supplementary Figure 1: Finite size effects of the hole polaron. Isosurface of CDFT weight function, showing that the distance between the third nearest neighbour of 5.9 Å is a factor of three smaller than the distance to the periodic image of this charge (15.4 Å).

Electronic coupling from ‘energy-gap’

Table 2 shows the electronic coupling calculated from CDFT (H_{ab}^{CDFT}) as well as from two alternative so-called ‘energy-gap’ methods: from the energy gap between the DFT transition state energy and CDFT energy

$$H_{\mathrm{ab}}^{gap1} = \frac{1}{2}(E_{CDFT}^{TS} - E_{DFT}^{TS}), \tag{1}$$

and from the HOMO-LUMO energy gap on the DFT transition state

$$H_{\mathrm{ab}}^{gap2} = \frac{1}{2}(E_{HOMO} - E_{LUMO}). \tag{2}$$

Table 2: Comparison of electronic coupling calculated with CDFT (H_{ab}^{CDFT}) as well as from energy gap between the DFT transition state energy and CDFT energy (H_{ab}^{gap1}) and from the HOMO-LUMO energy gap on the DFT transition state (H_{ab}^{gap2}).

		**Hole polaron**		
r / Å	Neighbour	H_{ab}^{CDFT} (meV)	H_{ab}^{gap1} (meV)	H_{ab}^{gap2} (meV)
2.97	$0^{(A)}$-$1^{(A)}$	203	44	183
	$0^{(A)}$-$1^{(B)}$	110	39	139
	$0^{(A)}$-$1^{(C)}$	101	38	137
	$0^{(B)}$-$1^{(B)}$	53	32	104
	$0^{(B)}$-$1^{(C)}$	39	31	93
	Average	147	40	152
		**Electron polaron**		
2.97	(0,1)-(0,2)	26	5	133
5.04	(0,2)-(1,6)	57	11	82
	(0,2)-(1,7)	19	8	71
	(0,1)-(5,11)	10	5	33

For the hole polaron it is found that the Boltzmann average electronic coupling calculated with CDFT and taken from the HOMO-LUMO gap are within numerical error (5 meV) of each other, despite the HOMO-LUMO gap method overestimating small couplings and underestimating high couplings relative to the CDFT value. The energy gap of the DFT transition state energy and CDFT energy however is insensitive to the coupling value, underestimating all coupling values.

For the electron polaron only the (0,2-1,6) coupling is important and therefore there is a greater dependence on the chosen method of electronic coupling method, 57 meV with CDFT and 82 meV with the HOMO-LUMO energy gap. The HOMO-LUMO energy gap method also interestingly gives a substantially larger coupling value for the smallest transfer distance (0,1)-(0,2), for which the small CDFT value is attributed to the 120 degree rotation of the t2g orbital which results in a small overlap of the two diabatic CDFT electronic wavefunctions and thus a small electronic coupling.

Calculation of average ET parameters

Boltzmann averages are used for combining the 9 possible transition states of the hole polaron, for both the reorganisation energy and the electronic coupling. These are calculated according to the following equations:

$$H_{\mathrm{ab}} = \left(\frac{\sum_i H^2_{\mathrm{ab,i}} e^{\frac{-\lambda_i}{4k_BT}}}{\sum_i e^{\frac{-\lambda_i}{4k_BT}}} \right)^{\frac{1}{2}}, \tag{3}$$

$$\lambda = 4 \left(\frac{\sum_i \frac{\lambda_i}{4} e^{\frac{-\lambda_i}{4k_BT}}}{\sum_i e^{\frac{-\lambda_i}{4k_BT}}} \right), \tag{4}$$

motivated from the form of the activation free energy

$$\Delta A^{\dagger} = \frac{\lambda}{4} - \left(\langle |H_{\mathrm{ab}}|^2 \rangle^{\frac{1}{2}}_{\mathrm{TS}} - \frac{\langle |H_{\mathrm{ab}}|^2 \rangle}{\lambda} \right), \tag{5}$$

where $\langle |H_{\mathrm{ab}}|^2 \rangle_{\mathrm{TS}}$ is the thermal average of the squared electronic coupling in the transition state (TS) and $\frac{\lambda}{4}$ is the activation free energy on the diabatic electronic states $\Delta A^{\ddagger}_{\mathrm{na}}$.

Table 3 shows a summary of the other possible combination methods: mean, root mean square and the Boltzmann average used in this work

Table 3: Comparison of methods used to calculate average hole coupling and average hole reorganisation energy. Activation energy, rate constant and 1D mobility are then calculated for these average values. All 9 possible transition states are accounted for in calculation of their average value. The 1D mobility is calculated using Equation 5 with i=2, accounting for the two nearest neighbours in a 1D chain of hematite.

Method	H_{ab} (meV)	λ (meV)	$\Delta E^{\ddagger}$ (meV)	k (s^{-1})	μ_{1D} ($\mathrm{cm}^2/\mathrm{Vs}$)
Mean	90	816	124	1.5×10^{11}	5.2×10^{-3}
Root Mean Square	102	818	115	2.1×10^{11}	7.3×10^{-3}
Boltzmann average	147	752	70	1.2×10^{12}	4.3×10^{-2}

Hole polaron structure

Table 4 shows the Hirshfeld spin moment and charge over the iron atom that the polaron localises. It can be seen that the difference in spin moment (+0.66 from neutral to hole ground state) is a good indicator of polaron formation while the change in charge (-0.02) is not. This is clearly visible in Figures 1-2, such that the change in spin density over the central iron atom is entirely positive (coloured yellow) while the change in electron density is both positive (yellow) and negative (blue).

Table 4: Charge and spin of iron atom that polaron localises from Hirshfeld analysis. 'Neutral GS' refers to neutral ground state, 'Hole' to neutral ground state geometry with an electron removed, 'Hole GS' to hole ground state and 'Neutral' to neutral state on the hole ground state geometry.

	Neutral GS	Hole	Hole GS	Neutral
Charge	0.44	0.44	0.42	0.40
Spin	-3.95	-3.93	-3.29	-3.86

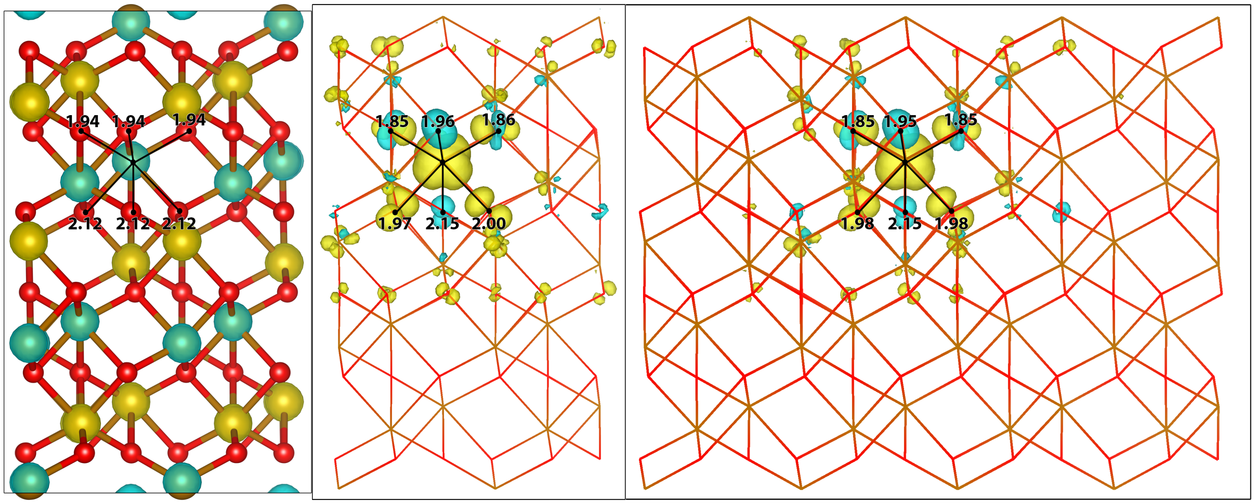

Supplementary Figure 1: 221 supercell spin density (left), 221 supercell spin density difference (middle) and 441 supercell spin density difference (right). Bond lengths between the iron site and bonded oxygen atoms are shown in Angstrom.

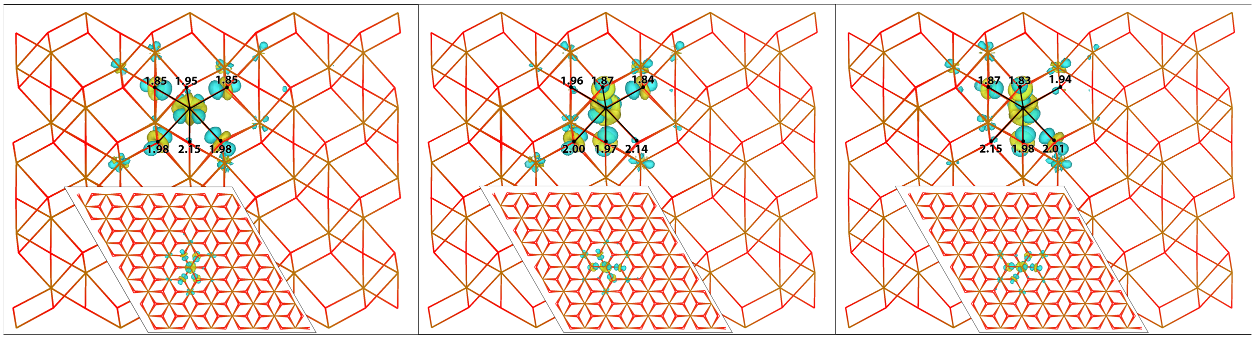

Supplementary Figure 2: 441 supercell electron density difference for ground state hole polaron. A hole polaron localised on an iron atom has three degenerate structures (A, B and C) due to the octahedral distortion of the Fe-O bonds. Bond lengths between the iron site and bonded oxygen atoms are shown in Angstrom.

Electron polaron structure

Table 5: Charge and spin of each iron atom that polaron localises from Hirshfeld analysis. 'Neutral GS' refers to neutral ground state, 'Electron' to neutral ground state geometry with an electron added, 'Electron GS' to electron ground state and 'Neutral' to neutral state on the electron ground state geometry.

	Neutral GS	Electron	Electron GS	Neutral
Charge	0.44	0.41	0.36	0.45
Spin	-3.95	-3.90	-3.73	-3.96

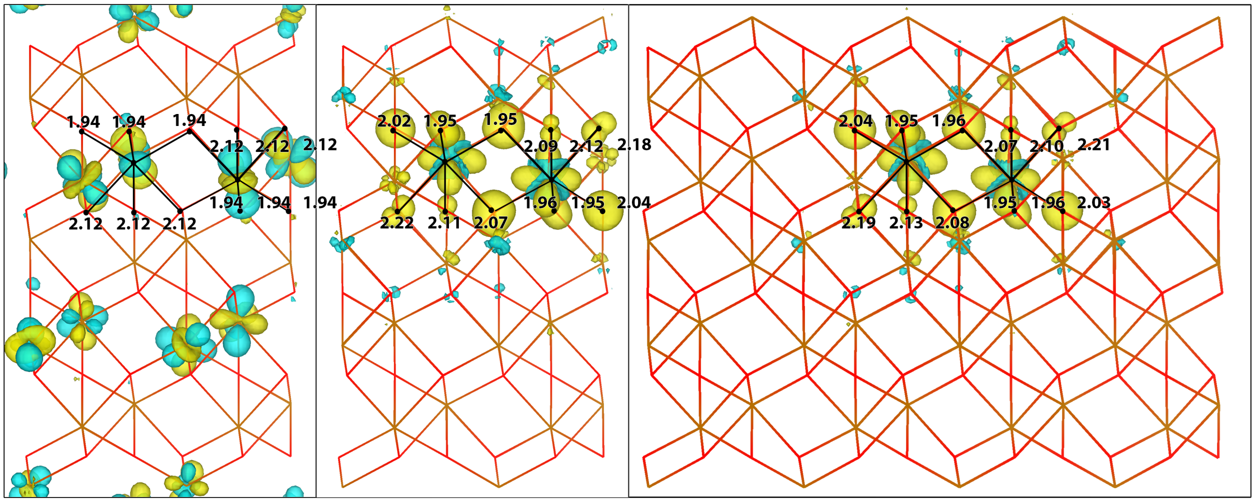

Supplementary Figure 3: 221 supercell HOMO from charged DFT (left), 221 supercell spin density difference (middle) and 441 supercell spin density difference (right). Bond lengths between the iron site and bonded oxygen atoms are shown in Angstrom.

Electron polaron transition states

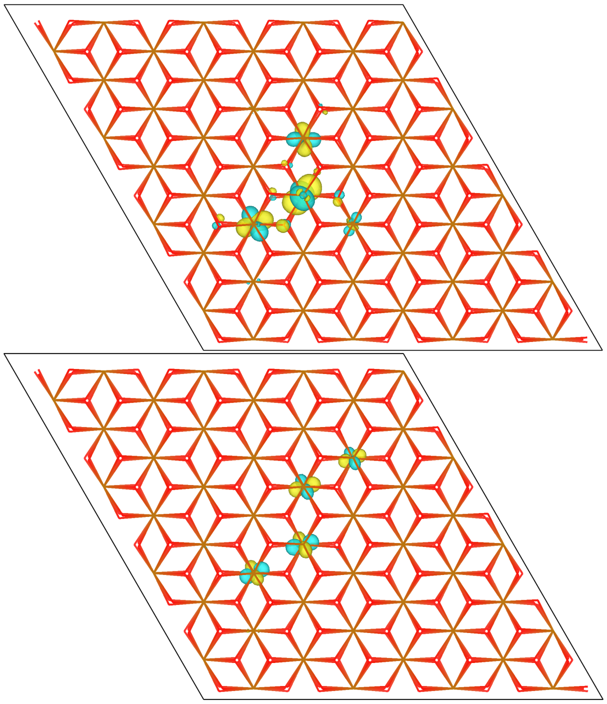

Supplementary Figure 4: Neutral LUMO for electron polaron transition state structures (0,1)-(0,2) (top left), (0,2) - (1,7) (top right), (0,2) - (1,6) (bottom left) and (0,1) - (5,11) (bottom right). Due to the transition state forming over up to four iron atoms, the LUMO provides a less visually distracting description of the transition state than the spin density difference.

Hopping schematic

Due to the 2-site delocalised electron polaron structure, there are only four symmetry related second nearest neighbours to which the polaron may hop (Supplementary Figure 5). This introduces a similar complication to the hole polaron, that for a single energy degenerate structure of the electron polaron the mobility is locally anisotropic. For ease of calculation, we consider the isotropic mobility for hopping to six second nearest neighbours and simply divide the final mobility by a factor of 4/6 to account for the four second nearest neighbours in the crystal.

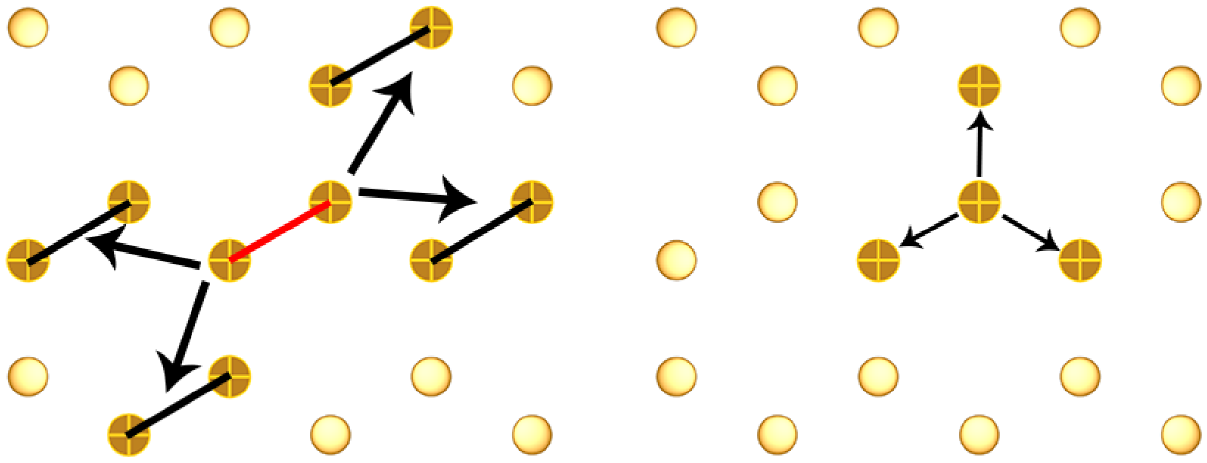

Supplementary Figure 5: 2D hematite plane showing the four highest coupling neighbours (5.04 Å) for the electron polaron (left), and three highest coupling neighbours (2.97 Å) for the hole polaron (right).

Mobility with re-calculated literature values

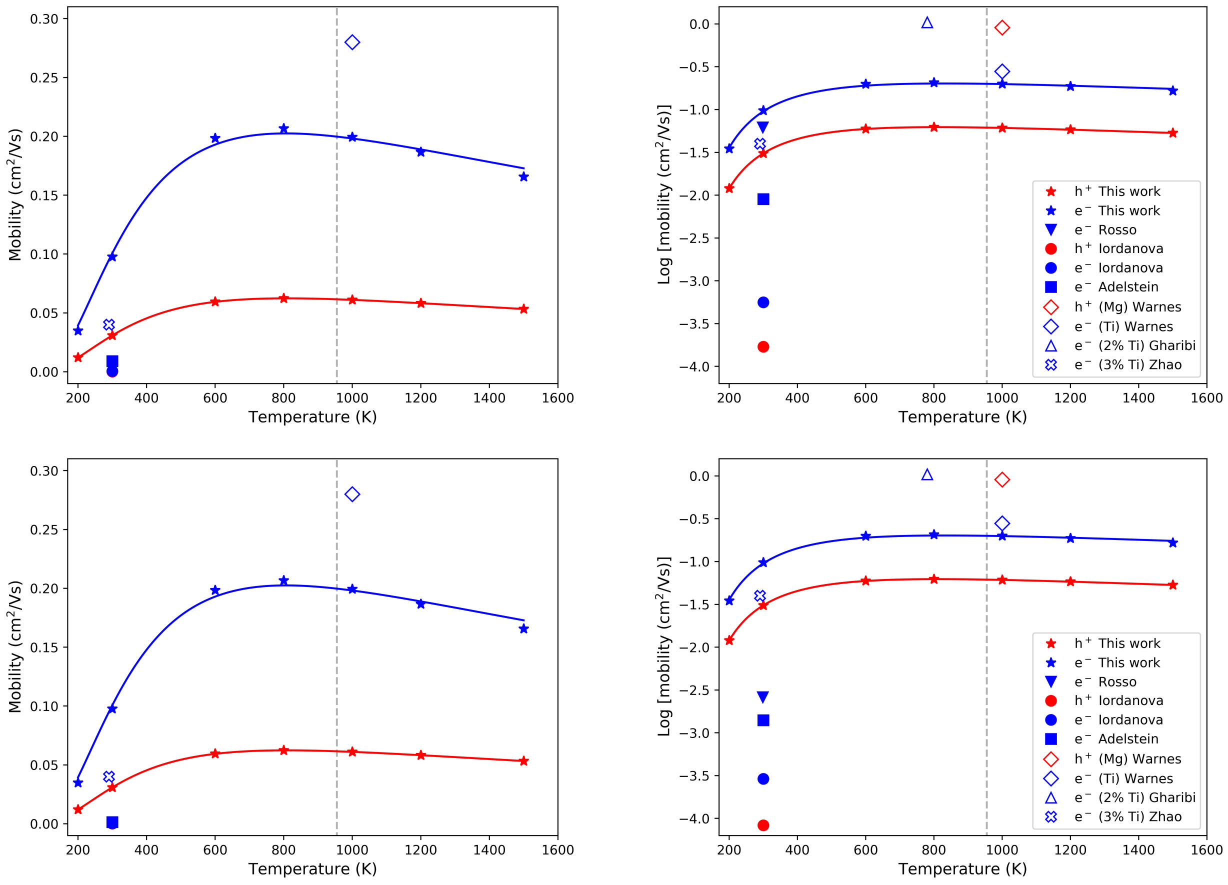

Supplementary Figure 6: Mobility as a function of temperature (left), and log mobility as a function of temperature (right). To facilitate direct comparison, literature calculated mobilities (top row) are re-calculated from provided couplings and reorganisation energies for our 2D plane (bottom row).

Overlap and coupling

We show the expected linear relationship between electronic coupling and overlap for the hole polaron.

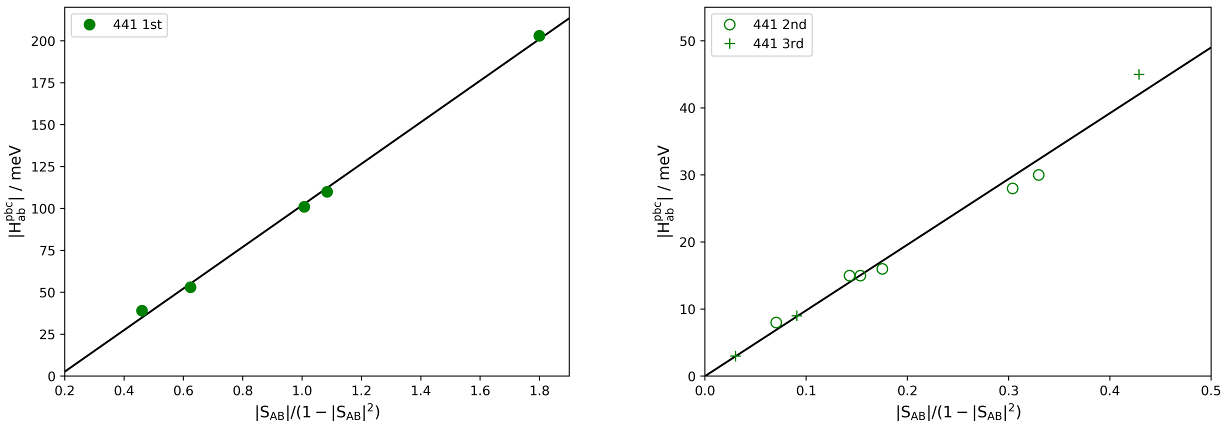

Supplementary Figure 7: Correlation plot between CDFT coupling and overlap for hole.

Implementation of Hirshfeld CDFT

We note that a modified version of the CP2K 8.1 code was used, with a complete implementation of Hirshfeld based CDFT including analytic forces. Code is available on request, and we intend to merge these changes into the current branch of CP2K in the future.
